# Calendar-Period Variation in the Association Between Documented Complete Rotavirus Vaccination and Laboratory-Confirmed Gastroenteritis: An 11-Year Test-Negative Study

**DOI:** 10.3390/pediatric18050127

**Published:** 2026-09-25

**Authors:** Yunus Nas, Basri Çakıroğlu

**Affiliations:** 1 Department of Pediatrics and Infectious Diseases, Hisar Hospital Intercontinental, 34768 Istanbul, Türkiye; dryunusnas@gmail.com; 2 Department of Urology, Hisar Hospital Intercontinental, 34768 Istanbul, Türkiye; 3 Deparment of Urology, Faculty of Medicine, Üsküdar University, 34768 Istanbul, Türkiye

**Keywords:** rotavirus, complete vaccination, test-negative design, odds ratio, acute gastroenteritis, hospitalization, children

## Abstract

Background/Objectives: Rotavirus vaccines have been available in Türkiye as self-financed products since 2006, but nationally representative uptake data and long-term data on their association with laboratory-confirmed rotavirus gastroenteritis remain limited. We evaluated calendar-period variation in the association between documented complete rotavirus vaccination and laboratory-confirmed rotavirus gastroenteritis among children younger than 5 years. Methods: This retrospective test-negative study included children aged 0–59 months who underwent qualitative stool rotavirus antigen testing during clinically evaluated acute gastroenteritis at a tertiary hospital between January 2015 and June 2026. Cases were rotavirus-positive episodes and controls were rotavirus-negative episodes. The institutional vaccination registry identified documented completion of either the two-dose Rotarix or three-dose RotaTeq schedule before testing, it did not separately identify partial schedules. Cluster-robust logistic regression adjusted for age group, sex, treatment setting, calendar year, and test month. Results are reported as odds ratios (ORs) with 95% confidence intervals (CIs). Results: The analysis included 2788 episodes among 2242 children: 534 rotavirus-positive cases and 2254 test-negative controls. Documented complete vaccination was recorded in 81 cases (15.2%) and 539 controls (23.9%). The adjusted OR for rotavirus positivity was 0.47 (95% CI, 0.36–0.62) for episodes with documented complete vaccination versus episodes with no documented complete series. Period-specific adjusted ORs were 0.85 (95% CI, 0.56–1.29) in 2015–2019, 0.64 (95% CI, 0.20–2.06) in 2020–2021, and 0.22 (95% CI, 0.13–0.38) in 2022–2026; between-period heterogeneity was significant (Q = 15.36; *p* < 0.001). The adjusted OR among hospitalized episodes was 0.42 (95% CI, 0.27–0.66). Among documented complete schedules, 448 episodes involved Rotarix and 172 involved RotaTeq; product-specific analyses were exploratory. Conclusions: The observed association varied substantially by calendar period; therefore, the pooled OR should not be interpreted as a stable national measure of vaccine performance. Within this tested clinical population, documented complete vaccination was associated with lower odds of laboratory-confirmed rotavirus gastroenteritis, including in the hospitalized test-negative analysis. Because the comparison group may have included partially vaccinated children, these findings are not conventional vaccine-effectiveness estimates. Nationally representative surveillance with complete dose-level vaccination histories is needed.

## 1. Introduction

Rotavirus remains an important cause of acute gastroenteritis, dehydration, emergency care, and hospitalization among young children. Although mortality has declined substantially in many settings, rotavirus continues to impose a clinically meaningful burden, particularly among children younger than 5 years. The World Health Organization recommends inclusion of rotavirus vaccination in national immunization programs as part of a comprehensive strategy for prevention and treatment of childhood diarrheal disease [1].

Randomized trials of the monovalent and pentavalent vaccines established high efficacy against severe rotavirus gastroenteritis [2,3,4]. Subsequent observational studies and meta-analyses confirmed large reductions in rotavirus-associated emergency visits, hospitalization, and mortality, while also showing that effectiveness varies across geographic regions, income settings, age groups, and outcome severity [5,6,7]. Recent hospital-based surveillance in the United States reported strong protection against emergency department visits and hospitalization and even higher effectiveness against very severe disease [8]. Before the availability of self-financed rotavirus vaccines, a multicenter prospective study documented the burden of rotavirus gastroenteritis in Türkiye [9].

Türkiye presents a distinct setting for vaccine-effectiveness evaluation. Both the monovalent Rotarix and pentavalent RotaTeq vaccines became available in 2006 as nongovernment-funded products and remain outside the routine national immunization schedule [10,11]. Vaccination is generally arranged through pediatric or family-medicine services in private or other healthcare institutions, with the vaccine obtained through pharmacies or the providing institution. Because families bear the cost, uptake may vary by household resources, community, access to private care, and clinician recommendation. At the time of revision, the listed retail price of one Rotarix dose was approximately TRY 3170, corresponding to more than TRY 6300 for the two-dose course before any administration charge [12]. Prices and product availability change over time. Although Türkiye has national electronic health and pharmaceutical tracking infrastructures, a complete, publicly available annual national series of self-financed rotavirus coverage was not identified; therefore, institutional uptake should not be interpreted as national coverage.

The test-negative design compares vaccination status between patients who seek care for the same clinical syndrome but differ in laboratory result and can reduce bias related to healthcare-seeking and access to testing [13,14]. Nevertheless, its validity depends on accurate exposure classification and it remains vulnerable to temporal changes in testing, residual confounding, and differential selection into the tested population.

These concerns are especially relevant to long study periods spanning the COVID-19 pandemic. Public-health interventions, changes in childcare attendance, altered healthcare-seeking, diagnostic practices, admission thresholds, and disrupted rotavirus circulation may change both the composition of tested patients and the observed association between documented vaccination and test positivity. Recent pediatric data from Türkiye and other settings demonstrate substantial pandemic-associated changes in rotavirus activity and clinical presentation [15,16,17].

The primary objective was to characterize calendar-period variation in the association between documented complete rotavirus vaccination and laboratory-confirmed rotavirus gastroenteritis among children aged 0–59 months. The pooled OR was treated as a secondary summary because the 11-year period spanned major epidemiological and healthcare disruptions. Additional objectives were to assess the association in hospitalized test-negative episodes, assess robustness under alternative episode and exposure definitions, describe vaccine product distribution, and evaluate whether complete vaccination was associated with hospitalization among breakthrough cases.

## 2. Materials and Methods

### 2.1. Study Design and Setting

This retrospective, single-center test-negative study was conducted at Hisar Hospital Intercontinental, a tertiary-care hospital in Istanbul, Türkiye. The study population comprised children aged 0–59 completed months who underwent stool rotavirus antigen testing during an acute gastroenteritis evaluation between 1 January 2015 and 30 June 2026. The cohort was a convenience sample of children presenting to this institution and tested at the treating physician’s discretion. A uniform testing protocol was not in place, and the database did not contain all acute gastroenteritis presentations without rotavirus testing; consequently, the proportion tested and differences between tested and untested children could not be determined. The report was prepared in accordance with the Strengthening the Reporting of Observational Studies in Epidemiology recommendations [18].

### 2.2. Data Sources and Linkage

Laboratory and clinical records provided the institutional protocol number, encounter number, sex, date of birth, test date, qualitative stool rotavirus antigen result, and inpatient or outpatient setting. The study extract did not retain the commercial assay manufacturer across the full 11-year period. A separate institutional vaccination registry contained children whose complete Rotarix or RotaTeq schedule had been administered or documented at this hospital, including vaccine product and completion date. It was not a countrywide registry and did not provide dose-level records for partial schedules. Laboratory, encounter, and vaccination records were linked deterministically, meaning that exact matches were made using the unique institutional protocol number rather than probabilistic matching. Sex was concordant in all linked records.

### 2.3. Eligibility and Episode Construction

Eligible records were rotavirus tests from children aged 0–59 months with a definitive positive or negative result and complete information on date of birth, test date, sex, and treatment setting. To avoid counting repeat testing during the same illness as independent observations, tests occurring within 14 days of a prior test in the same child were combined into one acute gastroenteritis episode. An episode was classified as positive if any test within the episode was positive; the earliest positive test was the index test for cases, and the first test was the index test for controls. Hospitalization was recorded when any encounter in the episode was inpatient. Alternative 7- and 30-day windows were examined in sensitivity analyses.

### 2.4. Case and Control Definitions

Cases were eligible acute gastroenteritis episodes with a positive stool rotavirus result. Controls were eligible episodes with a negative rotavirus result. Test-negative controls were drawn from the same institution, age range, study period, testing process, and clinical pathway as cases.

### 2.5. Vaccination Exposure

Documented complete vaccination was defined as an institutional record of an age-appropriate full course completed on or before the index test date: three doses of RotaTeq or two doses of Rotarix. The available registry extract contained completed schedules, product, and completion date but did not separately capture partial schedules. Accordingly, the comparator is described throughout as episodes with no documented complete series, rather than as confirmed unvaccinated episodes. Some children in the comparator could have received an incomplete series or vaccination outside the institution. The primary analysis combined the two documented complete schedules. A sensitivity analysis required completion at least 14 days before testing; product-specific analyses were exploratory because product use was correlated with calendar time.

### 2.6. Outcomes

The primary outcome was laboratory-confirmed rotavirus gastroenteritis. The principal secondary outcome was rotavirus-associated hospitalization, evaluated by restricting the test-negative analysis to hospitalized acute gastroenteritis episodes; within that analysis, hospitalized rotavirus-positive episodes were compared with hospitalized test-negative acute gastroenteritis episodes. A complementary case-only analysis included only the 534 rotavirus-positive episodes and compared hospitalization between children with a verified complete series and those without a documented complete series. Hospitalization was the only available severity indicator; validated severity scores, dehydration, intravenous-fluid use, intensive-care admission, and length of stay were unavailable. The data also did not establish whether the stool-test result was known before the admission decision.

### 2.7. Statistical Analysis

Continuous variables were summarized as medians and interquartile ranges (IQRs), and categorical variables as frequencies and percentages. Group comparisons used the Mann–Whitney U test for age and the chi-square or Fisher exact test for categorical variables, as appropriate.

Crude ORs were estimated from 2 × 2 tables. The primary multivariable logistic model included documented complete vaccination, age group (0–5, 6–11, 12–23, 24–35, and 36–59 months), sex, inpatient versus noninpatient setting, calendar-year fixed effects, and test-month fixed effects. Cluster-robust standard errors accounted for multiple episodes contributed by the same child. ORs and 95% CIs are reported directly; no vaccine-effectiveness calculation was performed.

Prespecified sensitivity analyses used 7- and 30-day episode windows, restricted the cohort to the first episode per child, required vaccination completion at least 14 days before testing, used a more parsimonious period-adjusted model, and analyzed hospitalized and nonhospitalized episodes separately. Calendar-period analyses for 2015–2019, 2020–2021, and 2022–2026 were emphasized because of expected pandemic-related disruption. Between-period heterogeneity was assessed using an inverse-variance Q statistic. Product-specific analyses compared each verified complete product schedule with episodes lacking any documented complete schedule and were considered exploratory. Two-sided *p* < 0.05 was considered statistically significant. Analyses were performed using Python version 3.12.0 with pandas version 2.0.0, NumPy version 2.0.0, and SciPy version 1.2.8.

### 2.8. Ethics

The study was conducted in accordance with the Declaration of Helsinki and approved by the Hisar Hospital Medical and Ethics Advisory Board on 31 July 2026 (Approval No. 26-77). The requirement for individual informed consent was waived because of the retrospective design and analysis of routinely collected, de-identified clinical data.

## 3. Results

### 3.1. Study Population

The database included 2849 rotavirus test records from 2242 children. Consolidating repeat tests within 14 days of the first test in an illness episode produced 2788 acute gastroenteritis episodes. Sixty-one repeat-test records were combined, and 10 episode groups contained discordant sequential results. The final cohort included 534 rotavirus-positive cases and 2254 test-negative controls (Figure 1).

A verified complete rotavirus vaccination series before testing was documented in 620 episodes (22.2%): 81 of 534 cases (15.2%) and 539 of 2254 controls (23.9%). Table 1 summarizes age, sex, treatment setting, verified vaccination status, age-group distribution, and calendar-period distribution. The proportion with a documented complete series varied across years from 11.1% to 35.2%; these values describe the tested institutional cohort and are not estimates of regional or national vaccine coverage.

### 3.2. Primary Association Analysis

Rotavirus positivity occurred in 81 of 620 episodes with documented complete vaccination (13.1%) and 453 of 2168 episodes with no documented complete series (20.9%). The crude OR was 0.57 (95% CI, 0.44–0.74; *p* < 0.001). After adjustment for age group, sex, treatment setting, calendar year, and test month, the pooled OR was 0.47 (95% CI, 0.36–0.62; *p* < 0.001). Because between-period heterogeneity was significant, this pooled OR is presented as a secondary summary rather than a stable estimate of association (Table 2; Figure 2).

### 3.3. Association in Hospitalized and Nonhospitalized Episodes

Among 1303 hospitalized acute gastroenteritis episodes, 241 were rotavirus positive and 1062 were test-negative controls. Documented complete vaccination was associated with lower odds of rotavirus positivity (adjusted OR, 0.42; 95% CI, 0.27–0.66; *p* < 0.001). Among 1485 nonhospitalized episodes, the adjusted OR was 0.48 (95% CI, 0.34–0.68; *p* < 0.001).

In the complementary case-only analysis restricted to 534 rotavirus-positive episodes, complete vaccination was not independently associated with hospitalization after adjustment (OR, 0.97; 95% CI, 0.51–1.82; *p* = 0.921). This finding indicates that the principal observed association was lower odds of rotavirus positivity among tested children, including among hospitalized presentations, rather than a difference in hospitalization conditional on breakthrough infection.

### 3.4. Sensitivity Analyses

The primary association was stable across alternative analytical definitions (Table 3). Requiring vaccination completion at least 14 days before testing yielded an OR of 0.46 (95% CI, 0.35–0.61). Restriction to the first episode per child yielded an OR of 0.45 (95% CI, 0.32–0.62). ORs using 7- and 30-day episode windows were 0.47 (95% CI, 0.36–0.62) and 0.47 (95% CI, 0.36–0.62), respectively. The parsimonious period-adjusted model produced an OR of 0.49 (95% CI, 0.37–0.64).

### 3.5. Calendar-Period Variation

Rotavirus positivity, testing volume, and documented completion varied substantially by year (Figure 3). The adjusted OR was 0.85 (95% CI, 0.56–1.29; *p* = 0.437) during 2015–2019, 0.64 (95% CI, 0.20–2.06; *p* = 0.454) during 2020–2021, and 0.22 (95% CI, 0.13–0.38; *p* < 0.001) during 2022–2026. Only 27 positive episodes occurred during 2020–2021, producing a very imprecise estimate. Between-period heterogeneity was significant (Q = 15.36; *p* < 0.001), indicating that a single pooled OR does not adequately represent the observed calendar-period variation (Table 4).

### 3.6. Vaccine Product Distribution

Among the 620 episodes with documented complete vaccination, 448 involved Rotarix (57 cases and 391 controls) and 172 involved RotaTeq (24 cases and 148 controls). Exploratory adjusted ORs were 0.49 (95% CI, 0.35–0.68) for Rotarix and 0.46 (95% CI, 0.28–0.75) for RotaTeq, each compared with episodes lacking a documented complete series. These estimates were directionally similar but should not be interpreted as a head-to-head comparison because product was correlated with calendar time and the comparator may include partial vaccination.

## 4. Discussion

### 4.1. Principal Findings

The principal finding was substantial calendar-period heterogeneity in the observed association. No statistically detectable association was found during 2015–2019, the 2020–2021 estimate was imprecise because few positive episodes occurred, and a strong inverse association was observed during 2022–2026. The pooled adjusted OR of 0.47 and the OR of 0.42 among hospitalized episodes summarize the full tested cohort but should not be interpreted as constant biological effects or nationally representative estimates.

The test-negative analysis materially changes the interpretation that would have arisen from studying positive cases alone. Within rotavirus-positive cases, documented complete vaccination was not associated with hospitalization after temporal adjustment. Nevertheless, when test-negative controls were included, documented complete vaccination was associated with lower odds of rotavirus positivity both overall and among hospitalized children. These results illustrate why case-only severity analyses cannot substitute for appropriately defined test-negative comparisons. A meta-regression of randomized trials has also shown that vaccine efficacy may vary by duration of follow-up [19].

The direction of the pooled association and the hospitalized analysis is consistent with international real-world evidence. Diallo et al. reported protection against rotavirus-associated emergency visits and hospitalization [8]. Gönüllü et al. studied the Turkish self-financed setting and likewise reported fewer rotavirus-positive and severe outcomes among vaccinated children [10]. Comparable associations have also been reported in a test-negative study from China and a real-world study from Spain [20,21].

The present study extends that work through an 11-year test-negative design, explicit period-specific ORs, sensitivity analyses, and a direct examination of temporal heterogeneity. However, the present estimates are not directly comparable with conventional vaccine-effectiveness estimates because the comparator may include partially vaccinated children.

We did not compare the ORs from this study numerically with conventional vaccine-effectiveness estimates reported elsewhere because their reference groups are not equivalent. Differences in outcome severity, age, testing selection, vaccination-record completeness, circulating strains, and healthcare access may contribute to differences across studies. The product-specific ORs were directionally similar, but they remain exploratory because Rotarix and RotaTeq use varied over calendar time and the institutional extract did not contain partial-series records.

### 4.2. Temporal Heterogeneity

The significant calendar-period heterogeneity is central to interpretation. The 2015–2019 OR crossed the null, the 2020–2021 OR was unstable, and the 2022–2026 OR was low. These differences may reflect changing rotavirus circulation, testing and admission thresholds, healthcare-seeking, childcare exposure, vaccine-record completeness, product mix, the mixture of partially and completely unvaccinated children in the comparator, or residual confounding across the COVID-19 era. They should not be interpreted as proof that biological vaccine performance changed over time.

Because the available variables cannot distinguish these mechanisms, the period-specific ORs are hypothesis-generating and context-dependent. For clinicians, the practical message is that documented complete vaccination was associated with lower rotavirus positivity in the overall tested population, but the magnitude cannot be generalized as a stable national effect or interpreted as conventional vaccine effectiveness. For policymakers, the findings support prospective multicenter surveillance linking standardized testing, complete dose-level national vaccination histories, socioeconomic measures, and genotype data.

### 4.3. Strengths

Strengths include a large pool of test-negative controls drawn from the same clinical pathway as cases, exact institutional identifier linkage, an 11-year observation period, complete age, sex, result, date, and treatment-setting data, and explicit adjustment for calendar year and month. Consolidation of repeat tests reduced serial-test duplication, child-clustered standard errors addressed multiple episodes, and sensitivity analyses were consistent with the pooled estimate. Product distribution and period-specific estimates are now reported transparently.

### 4.4. Limitations

This study has important limitations. First, the institutional vaccination extract identified documented completed Rotarix and RotaTeq schedules but did not separately capture partial schedules. Therefore, the comparator cannot be described as definitively unvaccinated; it includes episodes with no documented complete series and may include partially vaccinated children. Vaccination administered outside the institution could also have been missed. This heterogeneous comparator precludes a conventional vaccine-effectiveness estimate using a fully unvaccinated reference group. It may dilute the contrast between groups, but the magnitude and direction of bias cannot be guaranteed in the presence of differential documentation.

Second, the analysis lacked symptom-level information, validated Vesikari severity scores, dehydration grade, intravenous-fluid requirement, length of stay, intensive-care admission, comorbidities, socioeconomic variables, and childcare exposure. Hospitalization is clinically relevant but is also influenced by admission practices and healthcare access.

Third, testing was clinically indicated at the treating physician’s discretion rather than protocolized. The database did not include all acute gastroenteritis presentations without testing, so the proportion tested, systematic differences between tested and untested children, and whether vaccinated children were more likely to be tested could not be evaluated. The full 11-year extract also did not retain the commercial assay manufacturer. Changes in testing thresholds, laboratory implementation, outpatient access, referral patterns, and admission practices may have altered the composition of cases and controls. Year and month adjustment cannot eliminate this selection bias or residual temporal confounding.

Fourth, the data did not include circulating rotavirus genotypes. Genotype changes could contribute to period-specific variation.

Fifth, although completed schedules were defined according to the licensed product-specific regimens, product comparisons were exploratory. Among documented complete schedules, 448 episodes involved Rotarix and 172 involved RotaTeq. The adjusted exploratory ORs were 0.49 (95% CI, 0.35–0.68) and 0.46 (95% CI, 0.28–0.75), respectively, but these estimates should not be interpreted as a head-to-head comparison because product use was correlated with calendar time. Sixth, the study lacked nationally representative coverage, socioeconomic, and community-level data and cannot evaluate geographic or social inequity directly. Seventh, the single-center design limits generalizability. Finally, the study estimates association among children who presented and were tested, not protection against all community infections.

### 4.5. Clinical and Research Implications

Within the tested clinical population, documented complete rotavirus vaccination was associated with lower odds of laboratory-confirmed rotavirus gastroenteritis, including in the hospitalized test-negative analysis, but the magnitude depended strongly on calendar period. These ORs should not be interpreted as conventional vaccine-effectiveness estimates. Türkiye’s self-financed delivery model may create cost- and access-related inequities. National policy could improve equity by considering publicly funded inclusion in the routine immunization schedule, standardized recording of all doses regardless of provider, active monitoring of regional and socioeconomic uptake, and linkage of vaccination records with laboratory and hospitalization surveillance. These recommendations require confirmation in nationally representative implementation studies.

## 5. Conclusions

The association between documented complete rotavirus vaccination and laboratory-confirmed rotavirus gastroenteritis varied substantially across calendar periods, from no statistically detectable association in 2015–2019 to a strong inverse association in 2022–2026. Consequently, the pooled OR of 0.47 should be treated as a secondary, context-dependent summary rather than a stable national measure. Documented complete vaccination was also associated with lower odds of rotavirus positivity in the hospitalized test-negative analysis. Because the comparator may have included partially vaccinated children, these results must not be interpreted as conventional vaccine-effectiveness estimates. Nationally representative studies with standardized testing and complete dose-level vaccination records are needed to guide equitable policy in Türkiye.

## Figures and Tables

**Figure 1:**
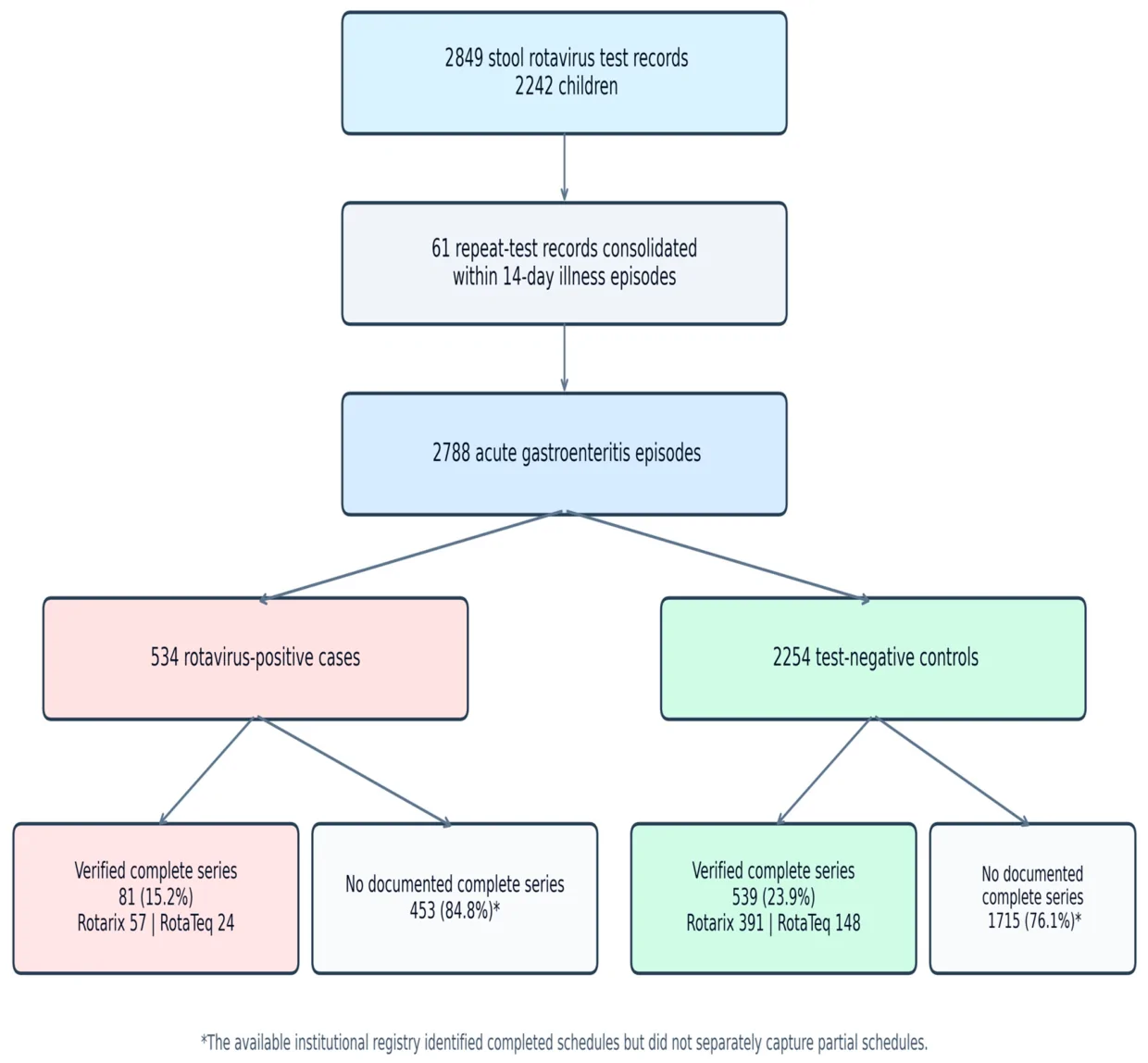
Study flow diagram showing source test records, 14-day episode consolidation, case–control classification, verified complete rotavirus vaccination by product, and episodes with no documented complete series. The institutional registry did not separately capture partial schedules. Blue boxes indicate the source population and consolidated episodes; red boxes indicate rotavirus-positive cases; green boxes indicate test-negative controls; and gray boxes indicate processing steps or episodes with no documented complete series.

**Figure 2:**
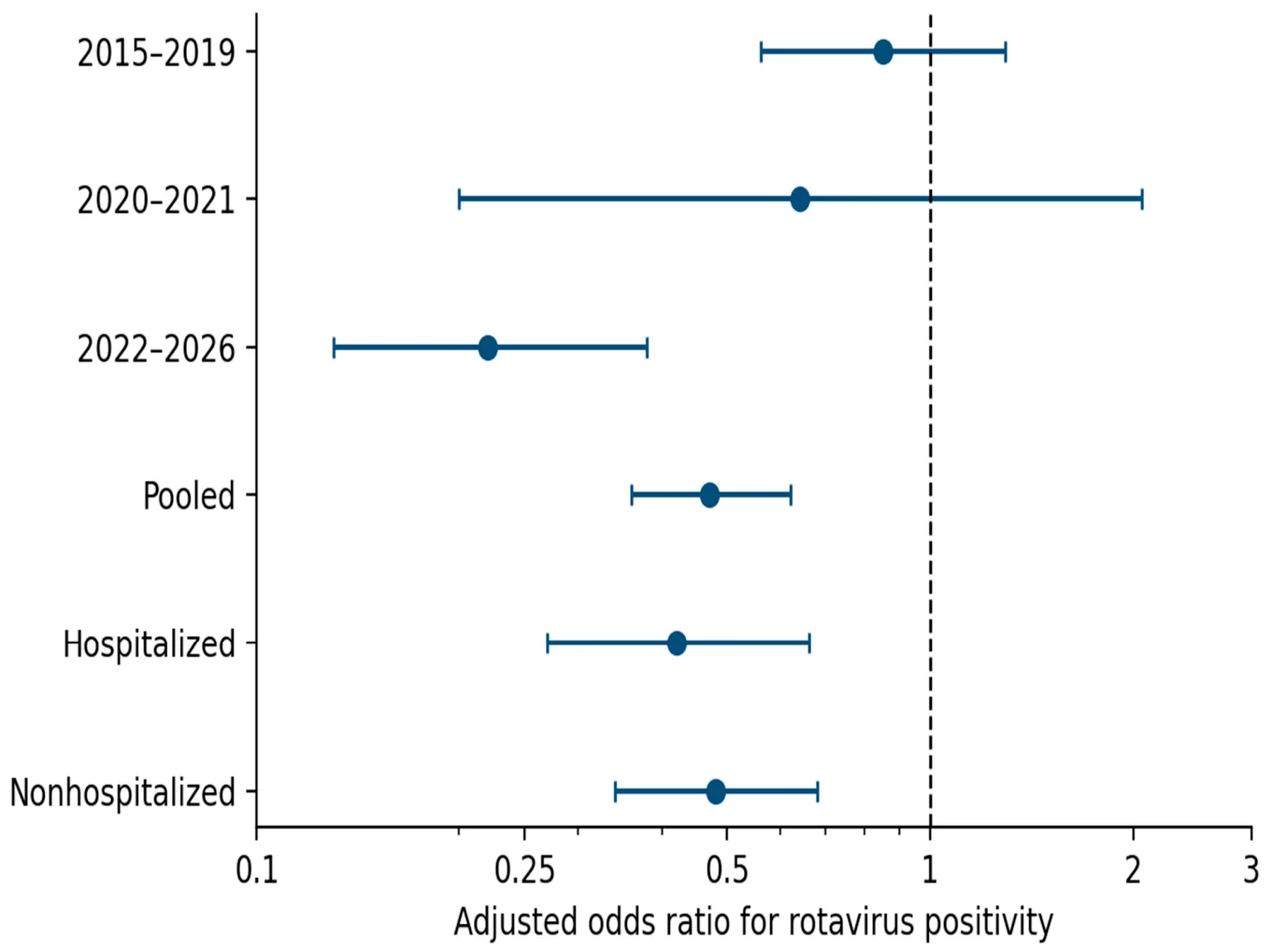
Adjusted period-specific, pooled, and treatment-setting odds ratios for rotavirus positivity comparing episodes with documented complete vaccination with episodes with no documented complete series. Points indicate ORs and horizontal lines indicate 95% CIs. The significant between-period heterogeneity (Q = 15.36; *p* < 0.001) limits interpretation of the pooled OR.

**Figure 3:**
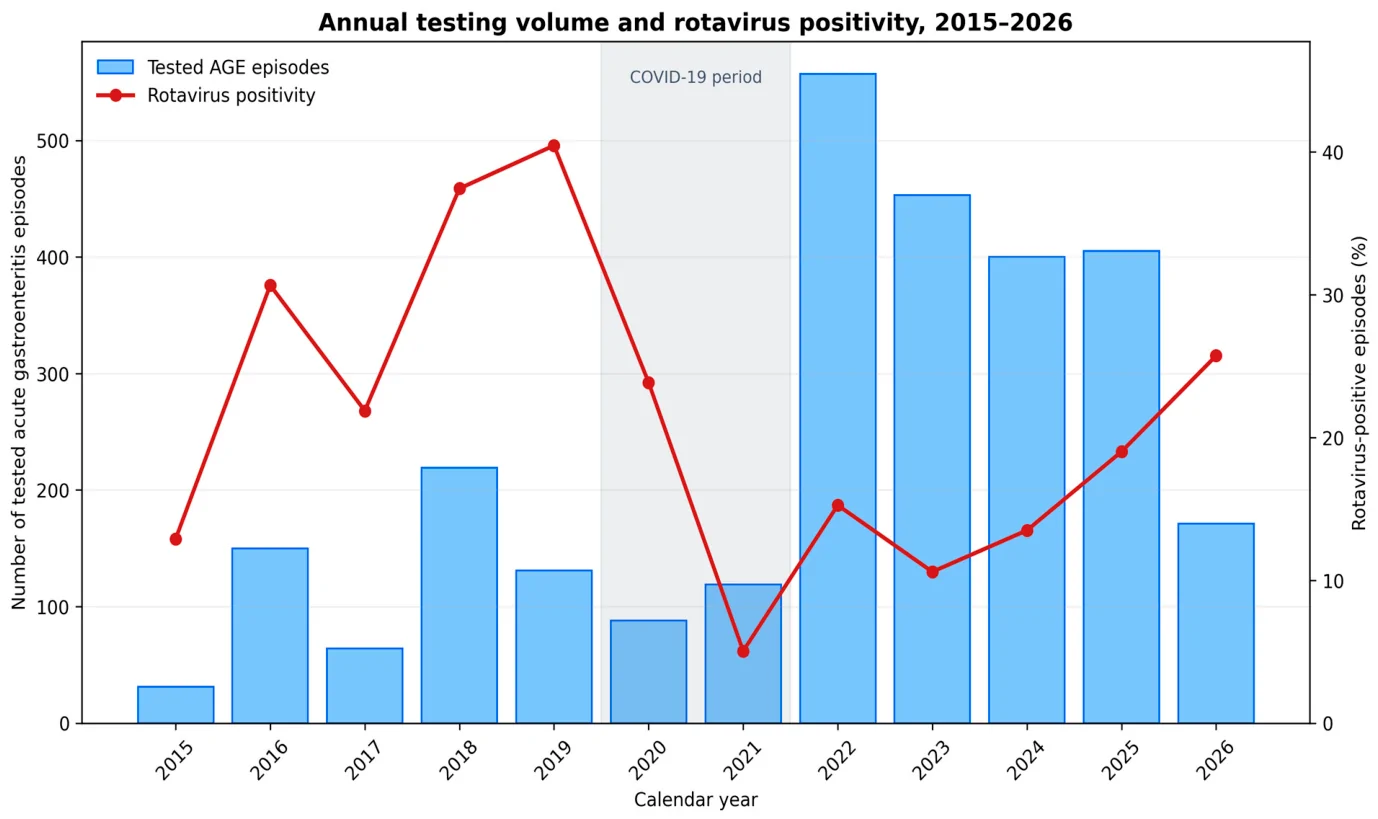
Annual numbers of acute gastroenteritis episodes tested and annual rotavirus positivity proportions during 2015–2026. Shading denotes the COVID-19 disruption period (2020–2021); the marked reduction in positivity in 2021 contributed to imprecision of the pandemic-period association estimate. Data for 2026 include January through June only.

**Table 1:** Characteristics of test-negative controls and rotavirus-positive cases.

Characteristic	Test-Negative Controls (*n* = 2254)	Rotavirus-Positive Cases (*n* = 534)	*p* Value
Age, months, median (IQR)	22.4 (11.0–38.9)	25.8 (14.9–40.2)	<0.001
Age group, months			<0.001
0–5	258 (11.4)	31 (5.8)	
6–11	372 (16.5)	65 (12.2)	
12–23	556 (24.7)	152 (28.5)	
24–35	409 (18.1)	121 (22.7)	
36–59	659 (29.2)	165 (30.9)	
Male sex	1233 (54.7)	284 (53.2)	0.558
Hospitalized	1062 (47.1)	241 (45.1)	0.436
Verified complete rotavirus series	539 (23.9)	81 (15.2)	<0.001
Calendar period			<0.001
2015–2019	396 (17.6)	199 (37.3)	
2020–2021	180 (8.0)	27 (5.1)	
2022–2026	1678 (74.4)	308 (57.7)	

**Table 2:** Odds ratios for laboratory-confirmed rotavirus gastroenteritis.

Analysis	Episodes	Cases	OR (95% CI)	*p* Value	*p* Value
Crude overall	2788	534	0.57 (0.44–0.74)	<0.001	<0.001
Adjusted overall	2788	534	0.47 (0.36–0.62)	<0.001	<0.001
Hospitalized episodes	1303	241	0.42 (0.27–0.66)	<0.001	<0.001
Nonhospitalized episodes	1485	293	0.48 (0.34–0.68)	<0.001	<0.001

**Table 3:** Sensitivity analyses.

Analysis	Episodes	Cases	Adjusted OR (95% CI)	*p* Value
Completion ≥14 days	2788	534	0.46 (0.35–0.61)	<0.001
First episode per child	2242	445	0.45 (0.32–0.62)	<0.001
7-day episode window	2811	534	0.47 (0.36–0.62)	<0.001
30-day episode window	2753	534	0.47 (0.36–0.62)	<0.001
Parsimonious period model	2788	534	0.49 (0.37–0.64)	<0.001

**Table 4:** Exploratory calendar-period analyses.

Calendar Period	Episodes	Cases	Documented Complete-Series Episodes	Adjusted OR (95% CI)	*p* Value
2015–2019	595	199	166	0.85 (0.56–1.29)	0.437
2020–2021	207	27	57	0.64 (0.20–2.06)	0.454
2022–2026	1986	308	397	0.22 (0.13–0.38)	<0.001

## Data Availability

The datasets generated and/or analyzed during the current study are available from the corresponding author upon reasonable request. The data are not publicly available because they contain information that could compromise patient privacy and confidentiality.
